# Supplementary material for: Investigating the influence of physiologically relevant hydrostatic pressure on CHO cell batch culture
Source: Sci Rep. 2021 Jan 8;11:162. doi: 10.1038/s41598-020-80576-8 (PMC7794228; doi:10.1038/s41598-020-80576-8)
Supplement: Supplementary file 1 — Supplementary Information 1. [file 41598_2020_80576_MOESM1_ESM.zip › S1/FLEX2/13Jan 30mmHg SS1 is C/Jan10 SS1.pdf]

===

Acq. Operator : SYSTEM Seq. Line : 5
Sample Operator : SYSTEM
Acq. Instrument : HPLC-0039 Location : 13
Injection Date : 1/14/2019 4:08:02 PM Inj : 1
Inj Volume : 100.000 µl
Acq. Method : C:\Users\Public\Documents\ChemStation\1\Data\TK_CHO_IgG 2019-01-14 15-49-10 \CHO_IgG_LC.M
Last changed : 12/4/2018 1:38:41 PM by SYSTEM
Analysis Method : C:\Users\Public\Documents\ChemStation\1\Data\TK_CHO_IgG 2019-01-14 15-49-10 \CHO_IgG_LC.M (Sequence Method)
Last changed : 1/14/2019 4:11:34 PM by SYSTEM
(modified after loading) (Current integration events modified)

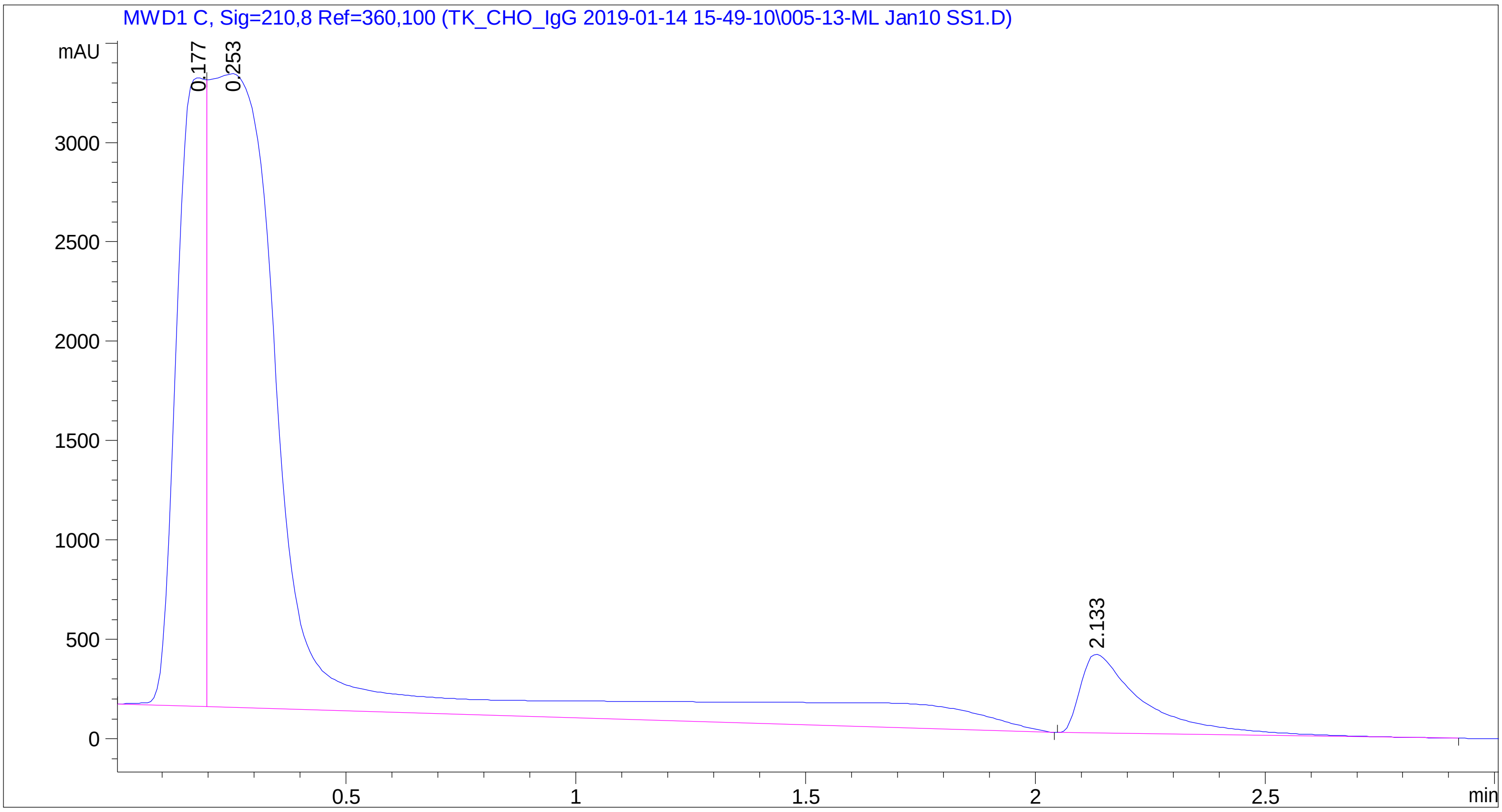

===

Area Percent Report

===

Sorted By : Signal
Multiplier : 1.0000
Dilution : 1.0000
Use Multiplier & Dilution Factor with ISTDs

Signal 1: MWD1 C, Sig=210,8 Ref=360,100

Peak #	RetTime [min]	Type	Width [min]	Area [mAU*s]	Height [mAU]	Area %
1	0.177	BV	0.0674	1.33990e4	3163.21411	23.7298
2	0.253	VB	0.1778	3.92909e4	3188.11255	69.5846
3	2.133	BBA	0.1331	3775.03442	396.50552	6.6856

Totals : 5.64650e4 6747.83218

===

*** End of Report ***
